# The complete chloroplast genome sequence of *Pohlia cruda* (Hedw.) Lindb.

**DOI:** 10.1080/23802359.2019.1693286

**Published:** 2019-11-21

**Authors:** Shijia Zhang, Yijie Zhang, Wenpan Dong, Jiangmin Wang, Miaoli Wu, Fengjiao Shen, Chao Xu, Jingyuan Niu, Lin Li, Shuo Shi, Jiancheng Zhao

**Affiliations:** a College of Life Sciences, Hebei Normal University, Shijiazhuang, China;; b State Key Laboratory of Systematic and Evolutionary Botany, Institute of Botany, Chinese Academy of Sciences, Beijing, China;; c School of Art and Design, Fuzhou University of International Studies and Trade, Changle, China;; d School of Basic Medical Sciences, Xinxiang Medical University, Xinxiang, China

**Keywords:** Bryales, Bryaceae, Mniaceae, Mielichhoferiaceae, bryophytes

## Abstract

*Pohlia* is a genus with many taxonomic and systematic controversies. In this study, the complete chloroplast genome of *Pohlia cruda* (Bryales, Bryophyte) was sequenced by high-throughput sequencing technology and described. The complete chloroplast genome is 125,114 bp in length and has a quadripartite structure. The two inverted repeat (IR) regions are 9921 bp long and separated by a large single-copy (LSC) region of 86,727 bp and a small single-copy (SSC) region of 18,545 bp. Phylogenetic trees were constructed based on the complete chloroplast genome sequences of 10 bryophytes downloaded from GenBank and one acquired in this study.

Bryales is one of the most important groups of globally distributed mosses (Guerra et al. 2011). However, the phylogenetic relationships among the families in Bryales have been controversial for the past few years (Goffinet et al. 2001; Niu et al. 2018). The controversy was mainly in the clade of Mniaceae-Mielichhoferiaceae, especially in the systematic position of the genus *Pohlia*. Brotherus (1903) placed *Pohlia* in Bryaceae based on morphological characteristics. However, the results based on chloroplast genome fragment sequences (Goffinet et al. 2001; Cox et al. 2014; Niu et al. 2018) showed that *Pohlia* was more closely related to Mniaceae than to Bryaceae and supported the transfer of *Pohlia* to Mniaceae. Shaw (2009) noted that there were distinct morphological differences among species of Mniaceae, such as species in the genera *Pohlia*, *Mielichhoferia*, and *Mnium*. Based on the results of morphological and molecular systematics, Hill et al. (2006) proposed the recognition of family Mielichhoferiaceae, represented by the genera *Mielichhoferia* and *Pohlia*.

In this study, the complete chloroplast genome sequence of *Pohlia cruda* (GenBank accession no. MN264338), as a representative of Bryales, was sequenced and described. The chloroplast genome of Bryales species has not been reported in the literature. This study will be very helpful for related studies of Bryales.

**Figure 1.**
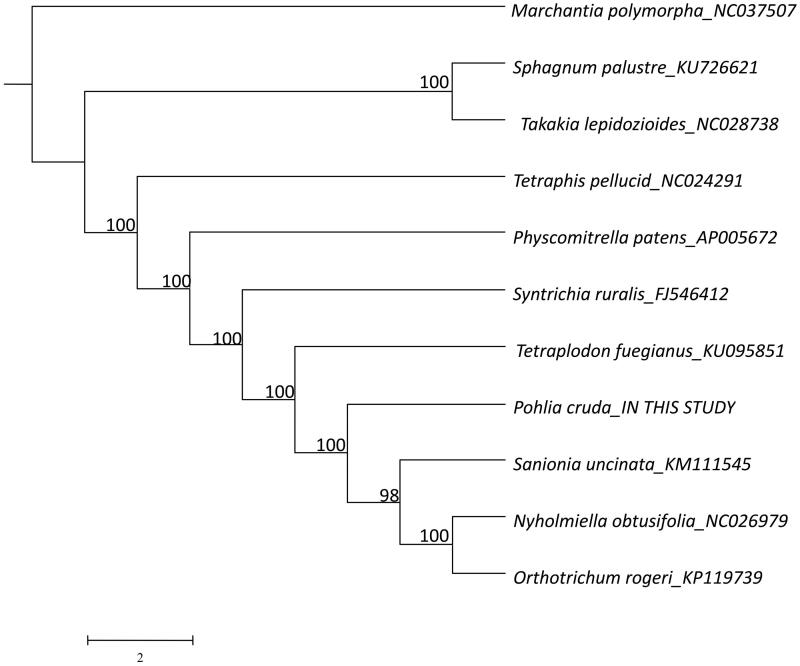
The ML tree based on 11 complete chloroplast genome sequences of bryophytes. Numbers on the branches are bootstrap values.

Genomic DNA was extracted from fresh leaves of *Pohlia cruda* by mCTAB method (Li et al. 2013). The specimen was collected from California and deposited in herbarium of Hebei Normal University, HBNU (specimen number: James 40450, herbarium barcode: HBNU050001). The Illumina HiSeq platform was used to sequence with a paired-end (PE) 150 genomic library (completed by Nuohezhiyuan Biotech Company, Beijing, China). A complete chloroplast genome of *Pohlia cruda* was assembled using the sequencing reads with Geneious (Kearse et al. 2012) and preliminarily annotated using DOGMA (http://dogma.ccbb.utexas.edu/) (Wyman et al. 2004). The final sequence information and gene annotation results were edited in Sequin v15.50.

The complete chloroplast DNA sequence of *Pohlia cruda* is 125,114 bp in length. The two inverted repeats (IRs) regions are 9921 bp separated by a large single-copy (LSC) region of 86,727 bp and a small single-copy (SSC) region of 18,545 bp. It contains 118 unique genes, including 82 protein-coding genes, 32 tRNA genes, and four rRNA genes. The overall G/C content for *Pohlia cruda* is 29.2%. There are 19 intron-containing genes, including eight tRNA genes and 11 protein-coding genes. The genes *ycf3* and *clpP* each contain two introns. The other 17 genes each have one intron. There are 149 simple sequence repeats (SSRs) in *Pohlia cruda* chloroplast genome.

To understand the phylogenetic relationships among selected moss species, an ML tree was constructed with PhyloSuite v1.1.14 (Zhang et al. 2018) with a TVM model based on the complete chloroplast genomes of 10 mosses and one liverwort (Figure 1, *Marchantia polymorpha*, as outgroup). All sequences were aligned using MAFFT v7.222 and manually adjusted with BioEdit v7.0.9.0. In the tree, *Pohlia cruda* formed a clade with the branch of Scorpidiaceae (*Sanionia uncinata*) and Orthotrichaceae (*Nyholmiella obtusifolia* and *Orthotrichum rogeri*) with high bootstrap support (100%).

## Geolocation information

This study was conducted and finished in College of Life Sciences, Hebei Normal University, No. 20, East Nanerhuan Road, Shijiazhuang, Hebei 050024, China.
